# A new species of the genus *Calliaxina* Ngoc-Ho, 2003 from the South China Sea (Crustacea, Decapoda, Axiidea, Callianassidae)

**DOI:** 10.3897/zookeys.635.10385

**Published:** 2016-11-23

**Authors:** Wenliang Liu, Xiaoli Liang

**Affiliations:** 1 Shanghai Key Lab for Urban Ecological Processes and Eco-Restoration, School of Ecological and Environmental Science, East China Normal University, Shanghai 200062, China; 2 The State Key Laboratory of Estuarine and Coastal Research, East China Normal University, East China Normal University, Shanghai 200062, China

**Keywords:** Callianassidae, Calliaxina, new species, South China Sea

## Abstract

A new species of the genus *Calliaxina* Ngoc-Ho, 2003, *Calliaxina xishaensis* **sp. n.**, collected from the South China Sea is described and illustrated. It is distinguishable from *Calliaxina thomassini* Ngoc-Ho, 2014 by having the rostrum broadly triangular with pointed tip and is distinguishable from *Calliaxina novaebritanniae* (Borradaile, 1900) and *Calliax punica* (de Saint Laurent & Manning, 1982) by the posterior margin of telson being convex. It is also the first record of this genus from the China seas. A key to the species of *Calliaxina* is given.

## Introduction

While working on the taxonomic study of the axiidean fauna (Crustacea, Decapoda) of the China Sea, an undescribed species assignable to the genus *Calliaxina* Ngoc-Ho, 2003 was found from Xisha islands, South China Sea. Ngoc-Ho (2003) established the genus *Calliaxina* mainly differing from *Calliax* de Saint Laurent, 1973 and *Eucalliax* Manning & Felder, 1991 by having an exopod on maxilliped 3. Sakai (2005) considered *Calliaxina* as a synonym of *Calliax*, but later Sakai (2011) recognised *Calliaxina* as valid: he argued that the presence of a sulcus was considered significant in the classification of the genera, and the presence of an exopod on the maxilliped 3 was not “of vital importance”, but he never-the-less expanded the genus to include eight species.

In this work, the classification of *Calliaxina* as defined by Ngoc-Ho (2003) is adopted, since the cardiac sulcus as an uncalcified suture running across the carapace between the cervical groove and the posterior border is regarded as significant, while the presence of an exopod on maxilliped 3 is a diagnostic character (Ngoc-Ho, 2014). Two species *Eucalliax kensleyi* Dworschak, 2005 and *Callianassa bulimba* Poore & Griffin, 1979 with rudimentary exopod on maxilliped 3 also should be belong to this genus.

Six species of the genus are known: *Calliax punica* (de Saint Laurent & Manning, 1982) is known from the eastern Atlantic and Mediterranean, and *Callianassa bulimba* (Poore & Griffin, 1979), *Callianassa kensleyi* (Dworschak, 2005), *Callianassa novaebritanniae* (Borradaile, 1900), *Callianassa sakaii* (de Saint Laurent & LeLoeuff, 1979), and *Callianassa thomassini* Ngoc-Ho, 2014 are known from the Indo-West Pacific.

### Key to the species of the genus *Calliaxina*

**Table:** 

1	Maxilliped 3 exopod rudimentary	**2**
–	Maxilliped 3 exopod distinct	**3**
2	Telson widest distally	***Calliaxina kensleyi***
–	Telson widest proximally	***Calliaxina bulimba***
3	Maxilliped 3 exopod short	***Calliaxina sakaii***
–	Maxilliped 3 exopod long, over-reaching ischium	**4**
4	Rostrum minute or nearly absent	***Calliaxina thomassini***
–	Rostrum broadly triangular with pointed tip	**5**
5	Posterior margin of telson convex	***Calliaxina xishaensis* sp. n.**
–	Posterior margin of telson almost straight	**6**
6	Telson 1.2 times as wide as long	***Calliaxina punica***
–	Telson 2.0 times as wide as long	***Calliaxina novaebritanniae***

## Methods

All specimens examined have been deposited in the Institute of Oceanology, Chinese Academy of Sciences, Qingdao, China (IOCAS). The drawings were made with the aid of drawing tube mounted on a Zeiss Stemi Sv11 compound microscope. The following abbreviation is used throughout the text: **MBM**: Marine Biology Museum; **CL**: carapace length.

## Taxonomy

### Family Callianassidae Dana, 1852 Genus *Calliaxina* Ngoc-Ho, 2003

#### Calliaxina xishaensis sp. n.

Taxon classificationAnimaliaDecapodaCallianassidae

http://zoobank.org/DC3F4E82-68C9-47EA-A894-B538F2FFEB3A

Figs 1 , 2

##### Material examined.

Holotype, ♀(cl, 7.0 mm), MBM136806/58C-639, Dengqin island of Xisha islands, South China Sea, coll. Zhengang Fan & Jieshan Xu, 11 May 1958. Paratype, ♀(cl, 5.8 mm), collected with holotype.

##### Diagnosis.

Rostrum broadly triangular with pointed tip, not reaching middle of eyestalks. Antennular peduncle over-reaching distal end of antennal peduncle penultimate segment. Maxilliped 3 exopod as long as ischium. Pereopods 1 subequal, slightly dissimilar. Left pereopod 1 cutting edge of fixed finger unarmed, with longitudinal depression scattered with small tubercles; right pereopod 1 cutting edge of fixed finger bearing large triangular tooth in basal 1/3 and with 14-17 small teeth from basal to subdistal. Telson 1.8 times as broad as long, poster margin convex.

##### Description.

**Figure 1.**
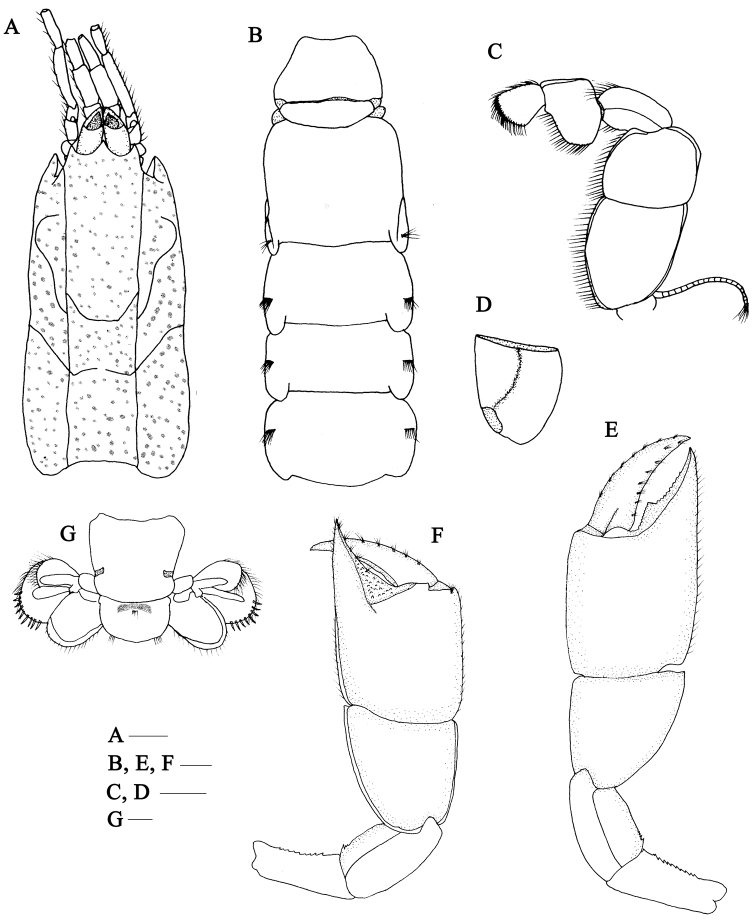
*Calliaxina xishaensis* sp. n. **A–F** Holotype female, MBM136806/58C-639 **A** carapace, dorsal and lateral view **B** pleomere, dorsal view **C** maxilliped 3, outer view **D** maxilliped 3, ischium, inner view **E** right cheliped, lateral view **F** left cheliped, lateral view **G** pleomere 6, telson and uropods. Scale bars: 1 mm.

Carapace scattered with small shallow depressions, lacking the dorsal oval. The frontal margin bears a broadly triangular rostrum, acute terminally, not reaching middle of eyestalks in dorsal view (Fig. 1A). Lateral projections produced, nearly reaching tip of rostrum. Cervical groove distinct, conjunct with linea thalassinica. Distinct suture (linea anomurica) ventral to hepatic boss extending posteroventrally to ventral margin of carapace. Cardiac suture in middle posterior half of carapace well defined, incomplete across midline of carapace, extending anterioventrally to linea anomurica.

Eyestalks dorsally flattened, approximately 1.5 times as long as broad at base, corneas rounded, pigmented subterminally, 0.7 width of eyestalks.

Antennular peduncle shorter but observably heavier than antennal peduncle (Fig. 1A); article 1 laterally and ventrally inflated; article 2 slightly longer than basal article, article 3 nearly 2/3 length of article 2; articles 2-3 with ventrolateral row of long, ventrally directed setae, continued onto ventral ramus of flagellum; rami of flagellum about equal length, nearly six times length of third article of peduncle; dorsal ramus with sparse short setae. Antennular peduncle (Fig. 1A) distinctly longer than antennal peduncle; article 1 with dorsolateral carina bearing regular line of fine setae above laterally produced excretory pore; article 3 shorter than article 2, with rudimentary scale on dorsal surface; article 4 elongate, longer than article 5 or combined length of first three.

Maxilliped 3 (Fig. 1C, D) exopod articulated, overreaching ischium of endopod. Ischium of endopod subtriangular, slightly longer than broad, medial longitudinally crista dentata on inner surface, holding 20-23 teeth; merus subquadrate about 0.8 time as long as broad; carpus strongly flexed in proximal third with setose lobe on lower margin; propodus subquadrate, 1.4 times broader than long; dactylus with rounded terminal border bearing dense closely set stiff setae.

Left and right pereopod 1 subequal, dissimilar in dentition of fixed fingers. Right cheliped (Fig. 1E) ischium slender, approximately 2.1 times as long as broad, upper margin almost straight, lower margin with 11-13 small denticles in middle. Merus about 1.8 times as long as broad, upper margin slightly convex, lower margin with 5-6 small denticles proximally. Carpus broad, increasing in breadth distally, lower margin arcuate, upper and lower margins keeled, terminating distally in triangular corners. Propodus heavy, 1.1 times as long as broad, inner surface of palm smooth; upper and lower propodal margins keeled, keel of lower becoming ill-defined beyond mid-length and absent on fixed finger, tufts of setae on inner face above lower margin; fixed finger thick, prehensile margin armed with one well-separated triangular tooth in mid-length, micro-serrations on upper margin of tooth and distally of it, distal 1/4 of finger unarmed, terminating in acute tip. Dactylus heavy, slightly longer than fixed finger, with pointed curved tip, unarmed.

Left cheliped (Fig. 1F) slightly smaller, similar in shape, cutting edge of fixed finger unarmed, but with a longitudinal triangular depression field of small tubercles on outer face; dactylus more slender than in major cheliped.

**Figure 2.**
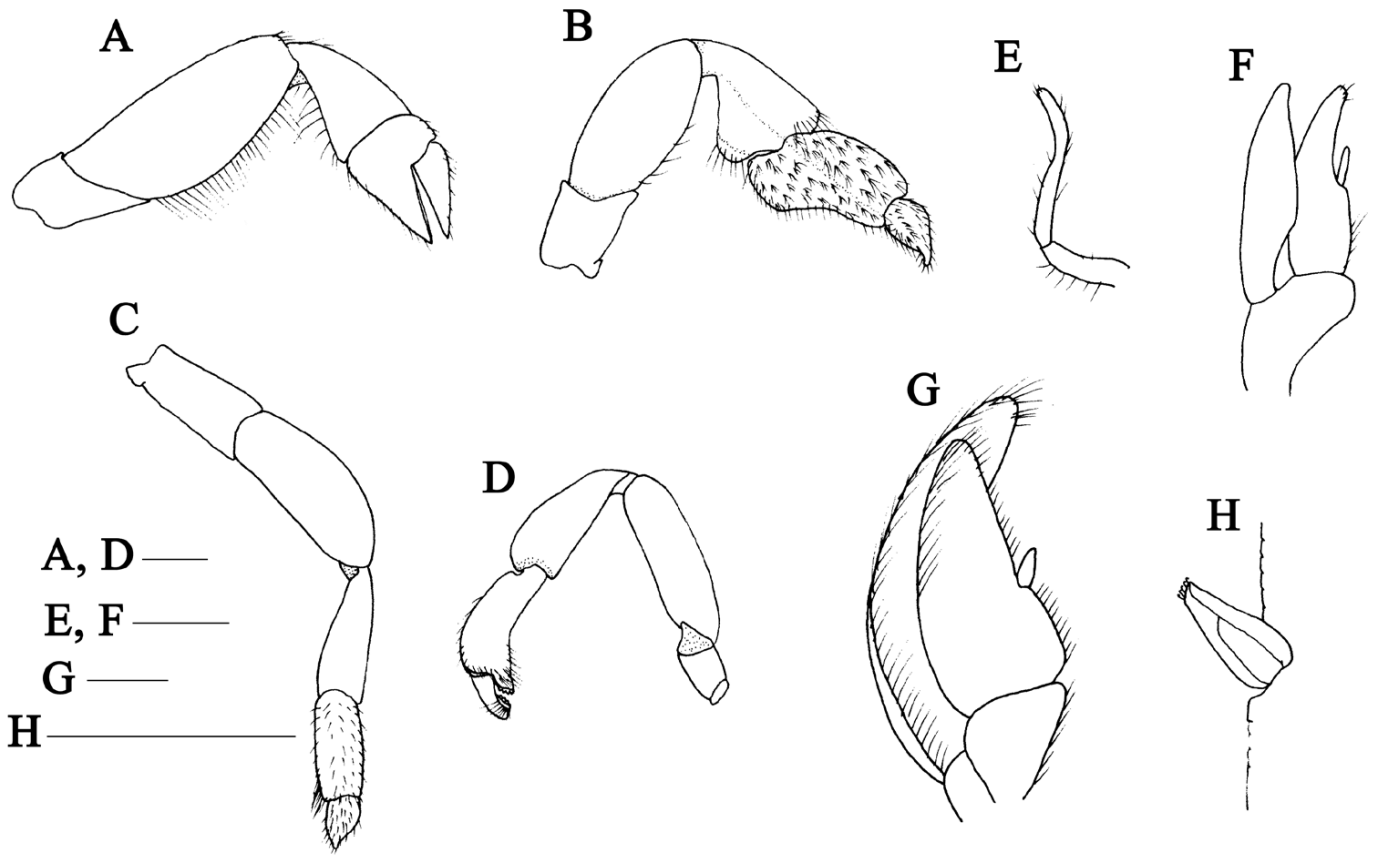
*Calliaxina xishaensis* sp. n. **A–H** Holotype female, MBM136806/58C-639 **A–D** pereopods 2-5, lateral views **E** pleopod 1, posterior view **F** pleopod 2, posterior view **G** pleopod 3, posterior view **H** appendix interna of pleopod 3, posterior view. Scale bars: 1 mm.

Pereopod 2 (Fig. 2A) ischium 0.7 times as long as high; merus 2.4 times as long as high, upper margin smooth, lower margin protruding and with row of dense long setae; carpus subtriangular, shorter than merus; chela shorter than carpus, with dense setae on lower and upper margins; palm with upper margin slightly convex; dactylus 2.8 times as long as upper margin of palm; carpus and chela fringed with short to long setae along margins.

Pereopod 3 (Fig. 2B) simple, moderately slender. Ischium slender, approximately as long as high; merus approximately 2.0 times as long as high, upper and lower margin slightly convex; carpus subtriangular, shorter than merus, broadest subdistally, approximately 1.4 times as long as high; propodus subrectangular, broadly flared distally to produce strong lower lobe, upper margin slightly convex and 0.7 length of carpus, with numerous tufts of setae on lateral surface and row of thick setae along upper and lower margins; dactylus subtriangular, upper and lower margins convex, outer surface densely setose, terminating in corneous tip.

Pereopod 4 (Fig. 2C) slender, all articles unarmed. Ischium rectangular; merus 1.6 times as long as ischium, upper margin convex; carpus 0.7 length of merus; propodus 0.7 length of carpus, lower margin densely setose; dactylus tapering distally, setose on lateral margin.

Pereopod 5 (Fig. 2D) minutely chelate, all articles unarmed. Ischium rectangular; merus nearly 4.3 times as long as ischium; carpus approximately 0.8 length of merus, upper margin swollen; propodus 0.6 length of carpus, lower distal corner projecting to form a chela with dactylus, lateral surface beset distally with dense setae; dactylus hooked excavate, spooned, toward external side of fixed finger, tips of dactylus and fixed finger obtuse.

Pleon long (Fig. 1B); dorsal length ratio (along midline) of first to sixth pleomere 1.0: 1.8: 1.2: 1.1: 1.0: 1.0. First pleomere narrowing anteriorly in dorsal view; dorsal tergite fused with the lateral pleurites; pleuron weakly developed but with clearly defined ventral margin. Second pleomere with concave anterior margin, posterior margin expanded posterolaterally, with two plumose setal rows near the posterior margin. Third to fifth pleomere each distinctly shorter than second somite; pleura each with tuft of moderately long plumose setae midlaterally and on posteroventral margin. Sixth pleomere subquadrate in dorsal view, very slightly narrowed posteriorly; lateral margin smooth, with a transparent, subrectangular punctae on posterior 1/4.

Female pleopod 1 (Fig. 2E) uniramous, of 2 articles, proximal article c. 0.5 length of distal article, long setae distally, distal article slender with a few setae. Female pleopod 2 (Fig. 2F) biramous, with appendix interna on endopod; exopod more slender than endopod. Pleopod 3–5 (Fig. 2G) biramous, foliaceous endopod bearing finger-like appendix internae (Fig. 2H).

Telson (Fig. 1G) c. 1.9 times as broad as long, broadest at midlength, posterolateral margin rounded, with one tuft of setae each near lateral margin, posterior margin convex; dorsal surface with row of long setae at anterior 1/4 and a transparent, banded punctae near anterior margin.

Uropodal endopod (Fig. 1G) subovate, longer than telson, 1.6 times as long as wide; margins unarmed; with distinct submedian carina on dorsal surface. Uropodal exopod (Fig. 1G) broad, fan-shaped, almost as long as wide, posterodistal margin with thick spiniform setae and dense fringe of setae; with distinct submedian carina and dorsal plate on dorsal surface, distal edge of carina lined with short spiniform setae.

##### Variation.

Maxilliped 3 exopod rudimentary in small specimen (paratype).

##### Etymology.

The species name is based on the type locality, Xisha islands, South China Sea.

##### Distribution and habitat.

Presently only known from the type locality.

##### Remarks.

The genus may be divided into three groups: maxilliped 3 with rudimentary exopod (*Callianassa bulimba* and *Calliaxina kensleyi*) and maxilliped 3 with distinct exopod (*Calliaxina novaebritanniae*, *Calliaxina punica*, *Calliaxina sakaii*, *Calliaxina thomassini*).

*Calliaxina xishaensis* sp. n. is the seventh species assigned to the genus. It is remarkably distinguished from the other species of the genus in the sixth pleomere somite with two lateral transparent, subrectangular punctae, and the dorsal surface of the telson with transparent, banded punctae.

The new species is closely related to *Calliaxina novaebritanniae* and *Calliaxina punica* in having the rostrum broadly triangular with a pointed tip, whereas *Calliaxina thomassini* has a minute or nearly absent rostrum. It can also be distinguished from *Calliaxina novaebritanniae* and *Calliaxina punica* by the convex posterior margin of the telson (versus straight). It is also similar to *Calliaxina bulimba* in the fixed finger of left cheliped bearing a longitudinal triangular depression field of small tubercles on its outer face, but It can be distinguished from latter by the distinct exopod on maxilliped 3 (versus rudimentary).

## Supplementary Material

XML Treatment for Calliaxina xishaensis

## References

[B1] Borradaile LA (1900) On the Stomatopoda and Macrura brought by Dr. Willey from the South Seas. In: Willey A (Ed.) Zoological results based on the material from New Britain, New Guinea, Loyalty Islands and elsewhere collected during the years 1895, 1896, and 1897. Cambridge, 395–428.

[B2] Dworschak PC (2005) A new species of *Eucalliax* Manning & Felder, 1991 (Decapoda: Callianassidae) from the Red Sea. Proceedings of the Biological Society of Washington 118: 209–217. doi: 10.2988/0006-324X(2005)118[209:ANSOEM]2.0.CO;2

[B3] Manning RB Felder DL (1991) Revision of the American Callianassidae (Crustacea: Decapoda: Thalassinidea). Proceedings of the Biological Society of Washington 104: 764–792.

[B4] Ngoc-Ho N (2003) European and Mediterranean Thalassinidea (Crustacea, Decapoda). Zoosystema 25: 439–555.

[B5] Ngoc-Ho N (2014) Six species of Axiidea and Gebiidea from the Indo-West Pacific (Crustacea, Decapoda). Zoosystema 36: 545–561. doi: 10.5252/z2014n3a1

[B6] Poore GCB Griffin DJG (1979) The Thalassinidea (Crustacea: Decapoda) of Australia. Records of the Australian Museum 32: 217–321. doi: 10.3853/j.0067-1975.32.1979.457

[B7] de Saint Laurent M (1973) Sur la systématique et la phylogénie des Thalassinidea: définition des familles des Callianassidae et des Upogebiidae et diagnose de cinq genres nouveaux. Comptes Rendus Hebdomadaires de Séances de l’Académie des Sciences (Paris) 277: 513–516.

[B8] de Saint Laurent M Le Loeuff P (1979) Campagnes de la Calypso au large des côtes Atlantiques Africaines (1956 et 1959) (suite). 22. Crustacés Décapodes Thalassinidea. I. Upogebiidae et Callianassidae. In: Forest J (Ed.) Résultats Scientifiques des Campagnes de la Calypso. Fasc. 11(22). Annales de l’Institut Océanographique, Monaco et Paris 55 suppl., 29–101.

[B9] de Saint Laurent M Manning RB (1982) *Calliax punica*, espéce nouvelle de Callianassidae (Crustacea, Decapoda) des eaux méditerranéennes. Quaderni della Laboratorio di Tecnologia della Pesca 3: 211–224.

[B10] Sakai K (2005) Callianassoidea of the world (Decapoda: Thalassinidea). Crustaceana Monographs 4: 1–285.

[B11] Sakai K (2011) Axioidea of the world and a reconsideration of the Callianassoidea (Decapoda, Thalassinidea, Callianassida). Crustaceana Monographs 13: 1–616.

